# Determinants of Flu Vaccine Uptake Among the General Population in Saudi Arabia: A Study Based on the Health Belief Model

**DOI:** 10.7759/cureus.41277

**Published:** 2023-07-02

**Authors:** Sulaiman I Alsuwailem, Ezzuddin A Okmi, Eid H Alkhaldi, Khalid S Almutairi, Waleed K Alshamari

**Affiliations:** 1 Preventive Medicine, Saudi Public Health Authority, Riyadh, SAU; 2 Respiratory Infectious Diseases Prevention and Control, Saudi Public Health Authority, Riyadh, SAU; 3 Preventive Medicine, Ministry of Health, Riyadh, SAU

**Keywords:** saudi arabia, public health, health belief model, determinants, flu vaccine uptake

## Abstract

Background and objective

Recently, influenza has emerged as a significant public health concern worldwide, including in Saudi Arabia. Vaccination against the flu is widely recognized as a crucial preventive measure to reduce morbidity and mortality associated with the virus. However, the uptake of flu vaccines among the general population in Saudi Arabia still remains low. In light of this, this study aimed to examine the determinants of influenza vaccine uptake in Saudi Arabia by using the Health Belief Model (HBM).

Methods

This cross-sectional study was conducted among adults living in all regions of Saudi Arabia by using an online self-administered questionnaire based on the HBM. The questionnaire inquired about demographics, knowledge about influenza, knowledge about vaccines, and beliefs/barriers. It was distributed via social media platforms, including WhatsApp, Twitter, and Instagram. IBM SPSS Statistics software version 29 (IBM Corp., Armonk, NY) was used for statistical analyses, and both the Chi-square test and logic regression analyses were applied to determine associations between explanatory and response variables, with the level of significance set at p<0.05.

Results

This study enrolled a total of 1040 participants, and the majority were Saudi nationals (96.9%). Of note, 66.2% of the participants were males, and the rest were females. Most of the participants were employed by governmental institutions (42.0%), had bachelor's degrees (58.4%), had never worked in the health sector (70.2%), and earned above 10,000 Saudi riyals per month (62.1%). Over half (55.7%) of participants had taken the flu vaccine at the time of this study. Working in the healthcare sector was associated with increased flu vaccine uptake [adjusted odds ratio (aOR): 3.84, p<0.001]. The likelihood of getting the flu vaccines was greater among men (aOR: 1.38, p=0.027), and obesity was associated with lower flu vaccine uptake (aOR: 0.29, p=0.034). Having contact with people with flu, having had flu in the past, and experiencing severe flu complications (aOR: 4.71, p=0.029; aOR: 0.13, p=0.006; and aOR: 0.29, p=0.033, respectively) were significantly associated with the flu vaccine uptake among our study participants. Perceived potential risks of the flu vaccine were also associated with taking the flu vaccine (aOR: 0.213, p=0.042). There was a significant association between seeing an advertisement for the flu vaccine and the likelihood of taking the vaccine (aOR: 5.488, p=0.042).

Conclusion

This study found that certain sociodemographic factors are associated with flu vaccine uptake. These factors included contact with flu-infected individuals, past experiences with flu, perceived risks, and exposure to flu vaccine advertisements. Improving healthcare accessibility, conducting awareness campaigns, and implementing workplace initiatives are recommended to address the issues related to flu vaccine uptake.

## Introduction

One of the major public health issues currently facing the world is influenza, an acute respiratory disease caused by influenza viruses [1]. According to the Centers for Disease Control and Prevention (CDC), two major types of influenza viruses exist: A and B [2]. Influenza A and B cause seasonal outbreaks of flu every year. Influenza A can be further classified into various subtypes based on its two main surface proteins - hemagglutinin and neuraminidase - while Influenza B has only one subtype [3]. According to the World Health Organization (WHO), influenza occurs worldwide, with an annual global incidence rate of 5-10% in adults and 20-30% in children [4].

Lower respiratory tract infection caused by influenza viruses is the fourth leading cause of death worldwide and the deadliest communicable disease [5]. Hence, vaccines are the most effective way of preventing the spread of the influenza virus and protecting people from its potentially severe consequences [6]. Studies analyzed in a systematic review demonstrated that vaccination rates within Asia are low, and most countries in Asia lack scientific research on vaccination behaviors [7]. In Saudi Arabia, research on vaccination coverage revealed a similar scenario. Alotaibi et al. found vaccine coverage in older people 65 years and above to be 47.8%, with at least one vaccination dose. Of those unvaccinated, 46% considered the vaccine to be unnecessary [8]. Additionally, Alwazzah et al. [9] revealed that 19.3% of 1734 patients diagnosed with coronavirus disease 2019 (COVID-19) had taken a seasonal influenza vaccine (SIV); the female and male coverage rate was 23.4 and 15.8%, respectively.

The effect of the COVID-19 pandemic on public health has been huge, and one of these effects was the growing vaccine hesitancy that accompanied the concerns about the safety of the newly developed COVID-19 vaccines. Hesitancy plays an essential role in the low coverage of SIV [10]. Vaccine hesitancy is considered one of the 10 threats to global health [11,12]. Arabian countries display the most heightened hesitancy rates compared to other parts of the world, with healthcare workers similarly hesitant as the general population [9]. Studies have shown that vaccine hesitancy was relatively low in Saudi Arabia, and Saudis had mostly positive attitudes [13-15]. Education, income levels (p=0.004), and the influence of the leader were found to be the factors related to this attitude among Saudi Arabians [14]. On the other hand, a study by Alamer et al. found that 24% of healthcare workers disagreed with the Saudi Ministry of Health (MoH)'s vaccination schedule, while 14% of them were unwilling to recommend or receive the vaccine in general, and 6% believed that the SIV strongly correlated with Guillain-Barré syndrome (GBS), and 8% thought there is an association between measles vaccine and autism [16]. Although vaccines have been proven to be safe and effective, hesitancy toward flu vaccination persists. One of the most practically used models for understanding vaccination behavior is the Health Belief Model (HBM), a commonly used framework in health behavior research developed in the 1950s [17,18]. According to HBM, individuals are more likely to get vaccinated if they perceive themselves to be at risk of the disease, see the disease as severe, believe the vaccine will protect them from contracting it or make symptoms less severe, and believe that the benefits of the vaccination outweigh any potential drawbacks [19]. HBM has been applied in several studies exploring vaccination acceptance rates for a variety of conditions [17].

To understand the determinants of flu vaccine uptake in Saudi Arabia, we used HBM to determine the rate of taking flu vaccines and identify the demographic and sociopsychological factors among adults associated with it. This study was conducted after the emergence of the COVID-19 pandemic, which has significantly affected perceptions toward vaccines, and its findings would be helpful to implement measures to ensure adequate flu vaccine uptake.

## Materials and methods

Study design

This cross-sectional study was conducted across all the principal regions of Saudi Arabia and involved adults aged 18 years and above living in Saudi Arabia.

Sample size and sampling technique

The sample size was calculated by using Raosoft (Raosoft Inc., Seattle, WA), and assuming a 50% response rate, 95% confidence interval (CI), Z-score of 1.96, and 5% margin of error, the minimum sample size determined was 385. Therefore, a minimum of 385 participants were needed to achieve a precision of ± 5% with 95% confidence. We used a snowball sampling technique to select 1040 prospective participants across all five regions.

Data collection tool

The initial draft of the questionnaire was developed in English and then translated into Arabic based on previous studies in the literature exploring vaccination acceptance. Two expert researchers with medical backgrounds reviewed the translated version and subsequently validated it.

The questionnaire consisted of four parts: part I inquired about the demographic data, part II addressed the knowledge about the influenza virus, part III involved the knowledge about the influenza vaccine, and part IV queried about the beliefs and barriers regarding the influenza vaccine. The questionnaire was distributed online on Google Forms, along with the invitation letter explaining the purpose of the study and the participant’s rights, and a consent form to sign before completing the questionnaire.

Statistical analysis

Descriptive analysis was used to describe the participants’ characteristics. Means and standard deviations (SD) were used for continuous variables, whereas frequencies and proportions were used for categorical variables. The dependent variable of interest was a willingness to receive the flu vaccine, and participants' responses to wiliness-related questions were in three categories (“Yes, Already took it,” “No,” or “Not sure”). For the analyses, we added the answer “No” to the answer “Not sure.” Then, the three responses were re-categorized into dichotomous variables “Yes” and “No.”

A univariate analysis using Chi-square testing identified candidate variables for the multivariate logistic regression at a p-value of <0.05 at a 95% confidence interval (CI) set as a cut-off point. Multivariate logistic regression analysis was applied to determine the association between multiple predictor variables and the dichotomized flu vaccine acceptance variable. No backward selection was used; only a variable with a significance level of p<0.05 remained in the model. All data analyses were performed using IBM SPSS Statistics software version 29 (IBM Corp., Armonk, NY).

Ethical considerations

Approval from the Institutional Review Board of King Fahad Medical City (Ref. no: 23-211E) was obtained. An informed consent statement was included on the initial page of the online questionnaire. All participants were informed that participating in the study would be voluntary. The questionnaire was anonymous, and the data for this study were kept confidential and used for the study’s purpose only.

## Results

**Figure 1:**
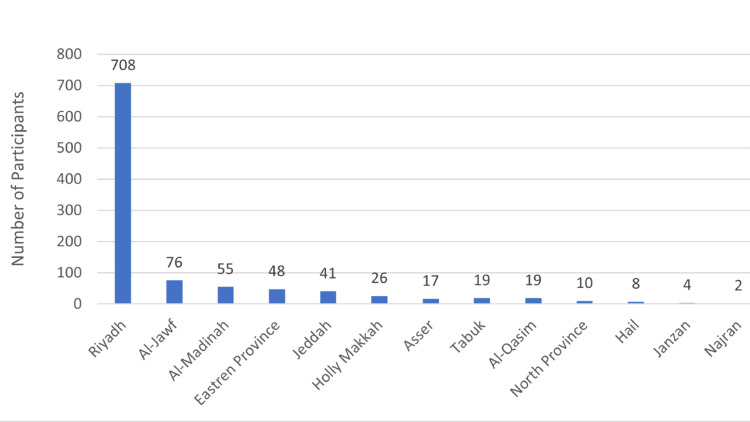
Proportion of participants from different regions of Saudi Arabia

Table 1 shows the sociodemographic characteristics of the participants in our study. The study included 1040 participants; the majority (66.2%) were male, and 33.8% were female. Most of them (66.4%) belonged to the age group of 21-40 years, followed by the 41-60-year age group (25.2%). The vast majority of participants were Saudi nationals (96.9%). Of all participants, 58.4% had a bachelor's degree, and 23.8% had higher education qualifications. Most participants (42.0%) were employed by governmental institutions, followed by those unemployed (28.9%) and 22.0% working in the private sector. The majority of the participants had never worked in the health sector (70.2%), while almost a third (29.8%) had prior experience working in healthcare. Income distribution among participants was as follows: 62.1%, 19.8%, and 18.1% of participants earned above 10,000 Saudi riyals, between 5,000 and 10,000 riyals, and below 5,000 riyals, respectively. The majority of respondents reported having no chronic diseases (81.8%). Over two-thirds of the participants resided in Riyadh, as indicated in Figure 1.

**Table 1:** Sociodemographic characteristics of participants (N=1040)

Characteristics		Frequency	Percent
Gender	Female	352	33.8
	Male	688	66.2
Age	<20 years	36	3.5
	21–40 years	691	66.4
	41–60 years	262	25.2
	61–80 years	50	4.8
	>80 years	1	0.1
Nationality	Non-Saudi	32	3.1
	Saudi	1008	96.9
Education	School	94	9.0
	Higher education	248	23.8
	Bachelor's degree	607	58.4
	Diploma	91	8.8
Occupation	Government job	437	42.0
	Military job	68	6.5
	Private sector job	229	22.0
	Unemployed	301	28.9
Ever worked in the health sector?	No	730	70.2
	Yes	310	29.8
Monthly income	<5k Saudi riyals	188	18.1
	5k–10k Saudi riyals	206	19.8
	>10k Saudi riyals	646	62.1
Chronic disease	No	851	81.8
	Yes	189	18.2

Table 2 presents the information on flu vaccine uptake as reported by our participants. Over half (55.7%) of the participants had taken the flu vaccine at the time of this study; while 37.9% had never taken it, 6.4% were not able to remember if they had taken it or not. Of those vaccinated, the highest proportion had taken it over a year ago (32.3%). Of all participants, most (61.1%) had no plan to take the flu vaccine in the year of this study, and of those who planned to get vaccinated that year, 10.8% did not have a specific timeframe in mind to get it done.

**Table 2:** Flu vaccine uptake among participants (N=1040)

		Frequency	Percent
Ever taken the flu vaccine?	Don't remember	67	6.4
	No	394	37.9
	Yes	579	55.7
If yes, when?	<3 months ago	42	4.0
	Between 3–6 months ago	84	8.1
	Between 6–12 months ago	115	11.1
	>1 year ago	336	32.3
Planning to get a vaccine this year?	No	635	61.1
	Yes	279	26.8
When do you plan to get the vaccine?	No specific timeframe	112	10.8
	Within 1 month	73	7.0
	Within 3 months	25	2.4
	Within 6 months	68	6.5

Table 3 presents the results of the binary logistic regression analysis examining sociodemographic determinants of taking the flu vaccine among participants. The analysis revealed that working in the health sector was significantly associated with higher flu vaccine uptake [adjusted odds ratio (aOR): 3.84, p<0.001]. Being male was also significantly associated with a higher likelihood of taking the flu vaccine (aOR: 1.38, p=0.027). However, being obese was associated with lower flu vaccine uptake (aOR: 0.29, p=0.034). The associations with other sociodemographic determinants were not statistically significant.

**Table 3:** Sociodemographic determinants of taking the flu vaccine (N=1040) P<0.05: statistically significant aOR: adjusted odds ratio; HTN: hypertension; DM: diabetes mellitus

Factors	B	P-value	aOR
Sex (male)	0.323	0.027	1.381
Nationality (Saudi)	0.057	0.884	1.059
Education (school)		0.359	Ref
Education (higher education)	0.205	0.478	1.228
Education (Bachelor's degree)	-0.081	0.734	0.922
Education (diploma)	-0.240	0.438	0.786
Occupation (govt.)		0.841	Ref
Occupation (military)	0.093	0.749	1.097
Occupation (private)	-0.061	0.711	0.941
Worked in the health sector	1.346	<0.001	3.844
Monthly income (<5k Saudi riyals)		0.142	Ref
Monthly income (5–10k Saudi riyals)	0.123	0.577	1.131
Monthly income (>10k Saudi riyals)	0.371	0.070	1.450
Chronic diseases	0.295	0.412	1.342
Asthma	0.231	0.629	1.259
HTN	0.036	0.932	1.037
DM	0.270	0.570	1.310
Obesity	-1.235	0.034	0.291
Constant	-0.643	0.182	0.526

**Figure 2:**
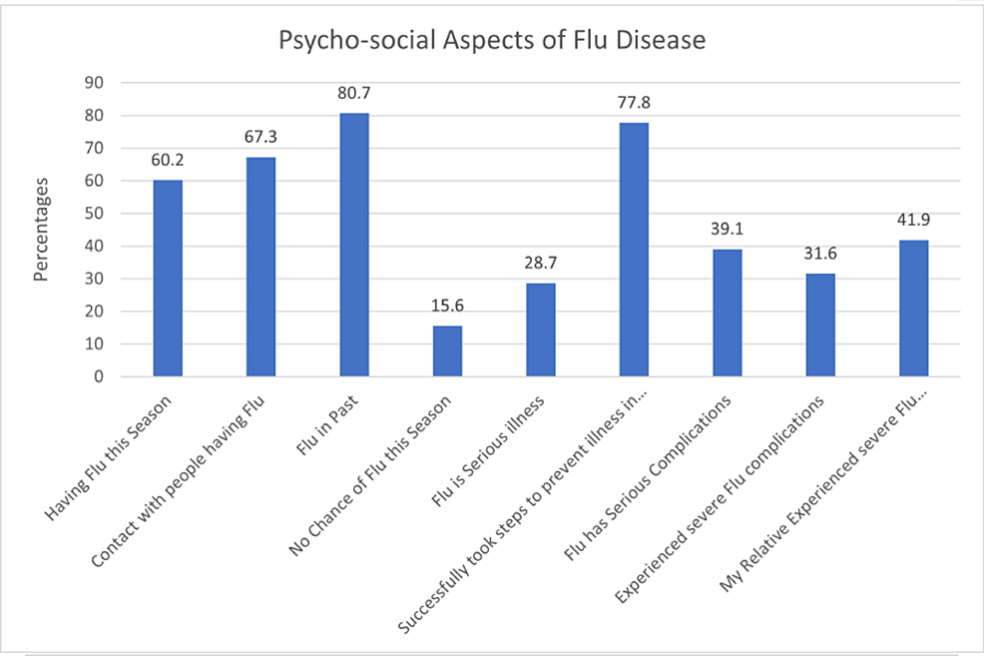
Proportions of participants in terms of psychosocial aspects of flu

Figure 2 depicts the psychosocial aspects of the flu as determinants for taking the flu vaccine. Most participants (60.2%) reported having the flu in the same season as the study period and having had flu in the past (80.7%). Over two-thirds (67.3%) of the participants had contact with people having flu, 39% believed that flu had serious complications, 31.6% reported having experienced flu complications, and 41.9% had relatives who had experienced flu complications. Over three-quarters (77.8%) reported having taken steps to prevent illness successfully, and 28.7% agreed that flu is a serious illness.

As shown in Table 4, contact with people having flu (aOR: 4.71, p=0.029), having had flu in the past (aOR: 0.13, p=0.006), and experiencing severe flu complications (aOR: 0.29, p=0.033) significantly influenced the flu vaccine uptake among our study participants. However, there was no statistically significant association between flu vaccine uptake and other psychosocial determinants analyzed.

**Table 4:** Psychosocial aspects of flu disease as determinants for taking the flu vaccine (N=1040) P<0.05: statistically significant aOR: adjusted odds ratio

		Frequency (%)	P-value	aOR
Having flu this season	Don't know	304 (29.2)	0.854	1.111
	No	111 (10.7)		
	Yes	625 (60.2)		
Contact with people having flu	Don't know	205 (19.7)	0.029	4.708
	No	135 (13.0)		
	Yes	700 (67.3)		
Flu in the past	Don't know	119 (11.4)	0.006	0.131
	No	82 (7.9)		
	Yes	839 (80.7)		
No chance of getting the flu this season	Don't know	349 (33.6)	0.133	0.366
	No	529 (50.9)		
	Yes	162 (15.6)		
Flu is a serious illness	Don't know	318 (30.6)	0.576	0.726
	No	423 (40.7)		
	Yes	299 (28.7)		
Successfully took steps to prevent illness in the past	Don't know	142 (13.7)	0.785	0.826
	No	89 (8.6)		
	Yes	809 (77.8)		
Flu has serious complications	Don't know	328 (31.5)	0.849	1.131
	No	306 (29.4)		
	Yes	406 (39.0)		
Experienced severe flu complications	Don't know	139 (13.4)	0.033	0.285
	No	572 (55.0)		
	Yes	329 (31.6)		
My relatives have experienced severe flu complications	Don't know	239 (23.0)	0.169	0.416
	No	365 (35.1)		
	Yes	436 (41.9)		
Constant			0.001	383.924

**Figure 3:**
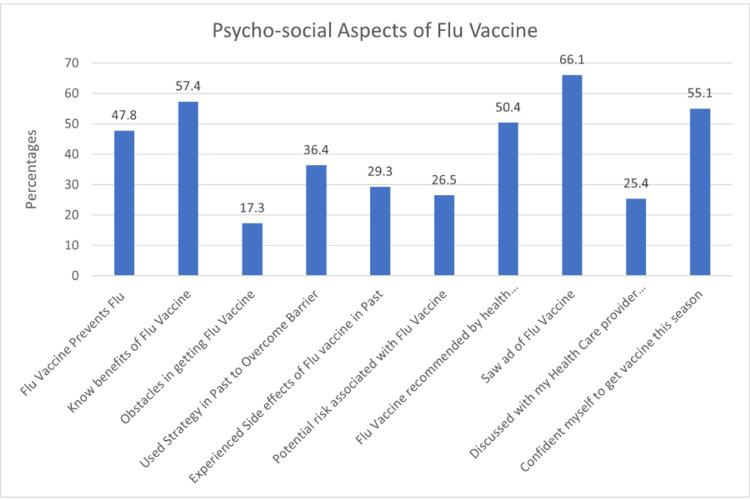
Proportions of participants in terms of psychosocial aspects of vaccine-related parameters as determinants of taking the flu vaccine

Figure 3 presents psychosocial aspects of vaccine-related parameters as determinants of taking the flu vaccine. Regarding the perception that the flu vaccine prevents flu, 47.8% agreed, and 57.4% reported knowing the benefits of the flu vaccine. Over two-thirds (66.4%) reported no obstacles in getting the vaccine. The analysis revealed a non-significant association between perceiving obstacles in getting the flu vaccine and the likelihood of taking the vaccine, with a higher aOR of 2.064. Almost a third (29.3%) of the participants had experienced side effects from the flu vaccine in the past, and another third (31.6%) had not. Almost a quarter of the participants (26.5%) believed that there were risks associated with flu vaccines, while another quarter (25.8%) did not. Half (50.4) of the participants had received a recommendation for the flu vaccine from a healthcare professional, and 66% had seen an ad for the flu vaccine. Over half (54.4%) of participants reported discussing the flu vaccine with their healthcare provider, and 55.2% reported feeling confident to take it in the current season.

As shown in Table 5, the analysis revealed a significant association between perceiving potential risks and the likelihood of taking the flu vaccine (aOR: 0.213, p=0.042). There was also a significant association between seeing an ad for the flu vaccine and the likelihood of taking the vaccine (aOR: 5.488, p=0.042). The associations with other vaccine-related parameters were not statistically significant.

**Table 5:** Psychosocial aspects of vaccine-related parameters as determinants of taking the flu vaccine P<0.05: statistically significant aOR: adjusted odds ratio

		Frequency	P-value	aOR
The flu vaccine prevents the disease	Don't know	392 (37.7)	0.861	0.863
	No	151 (14.5)		
	Yes	497 (47.8)		
Know the benefits of the flu vaccine	Don't know	311 (29.9)	0.216	0.307
	No	132 (12.7)		
	Yes	597 (57.4)		
Obstacles in getting the flu vaccine	Don't know	169 (16.3)	0.296	2.064
	No	691 (66.4)		
	Yes	180 (17.3)		
Used strategy in the past to overcome barriers	Don't know	390 (37.5)	0.986	1.012
	No	271 (26.1)		
	Yes	379 (36.4)		
Experienced side effects of the flu vaccine in the past	Don't know	193 (18.6)	0.055	0.349
	No	329 (31.6)		
	Yes	305 (29.3)		
There are potential risks associated with the flu vaccine	Don't know	392 (37.7)	0.042	0.213
	No	268 (25.8)		
	Yes	380 (26.5)		
Flu vaccine recommended by health professionals	Don't know	182 (17.5)	0.593	1.502
	No	334 (32.1)		
	Yes	524 (50.4)		
Saw an ad for the flu vaccine	Don't know	138 (13.3)	0.040	5.488
	No	216 (20.8)		
	Yes	686 (66.0)		
Discussed the vaccine with my healthcare provider	Don't know	210 (20.2)	0.126	0.338
	No	566 (54.4)		
	Yes	264 (25.4)		
Confident to get the vaccine this season	Don't know	274 (26.3)	0.992	1.008
	No	193 (18.6)		
	Yes	573 (55.1)		

## Discussion

After the emergence of the COVID-19 pandemic, the Saudi MOH has urged citizens and residents to take the flu vaccine to reduce the burden of the flu on the nation’s hospitals and clinics, which were already overloaded. The MOH recommended universal flu vaccination to citizens. However, the uptake of flu vaccines among the general population remains low in Saudi Arabia, despite the importance of vaccination in preventing influenza-related illnesses and complications [20]. This study explored the various factors that contribute to the decision-making among individuals regarding flu vaccine uptake in the country. By understanding these determinants, we can identify and implement strategies to promote higher vaccination rates.

We found that only slightly more than a half (55.7%) of the participants had taken the flu vaccine at least once at the time of this study, which is still low but almost similar to the flu vaccination rate reported by previous studies, which ranges from 12.7% to 55.0% [21-24]. Although Saudi Arabia has made progress in expanding its healthcare infrastructure, factors such as geographic location, socioeconomic status, and cultural norms can still create barriers to accessing vaccination centers and taking the vaccine. These factors might lead to a lower sense of willingness to get vaccinated, as evidenced by our finding that 61.1% had no plan to take the flu vaccine in the year of this study. Steeped in Islamic traditions, Saudi Arabia needs to take extra and unique efforts to ensure vaccine acceptance among its citizens due to cultural and religious beliefs strongly influencing health behaviors [13]. Collaborating with religious leaders, providing transparency with regard to vaccine production, and offering Halal-certified vaccines can address concerns related to cultural and religious beliefs.

Our findings showed that working in the health sector and being male were significantly associated with higher flu vaccine uptake rates (p<0.001 and p=0.027, respectively). Studies have indicated that people with higher knowledge about flu vaccines were significantly more likely to get SIV regularly, which explains the higher uptake among healthcare workers [8,20]. This highlights that the level of health education and awareness is crucial in influencing flu vaccine uptake. More comprehensive public health campaigns are needed to disseminate accurate information about the flu and address misconceptions about vaccine safety and efficacy. Utilizing social media platforms and engaging healthcare professionals as advocates can enhance awareness and build confidence in the flu vaccine. Some studies have indicated that more males (45.6%) than females (43.6%) showed a slightly higher readiness to receive vaccinations [25-27], while others have reported a higher uptake among male than female participants in contrast to other previous studies that found higher uptake among females (p<0.001) [28]. While obesity is a risk factor for complications from influenza infections [29], we found that it was associated with lower flu vaccine uptake (aOR: 0.29, p=0.034), which aligns with some studies that reported reduced influenza vaccination uptake in patients with morbid obesity [30]. In addition to obesity, it was found that patients with chronic liver illness and splenic dysfunction are less likely to take vaccinations in the United Kindom [31]. A study conducted in Italy among obese participants found that younger age, a medium level of education, the absence of a chronic illness, a smoking habit, and a claim that they had no contact with a doctor in the previous year were barriers to taking the influenza vaccination [29]. Similar demographic characteristics were highlighted among our participants: most were young (21-40 years old), 58.4% had a bachelor's degree, and 81.8% had no chronic diseases.

The study's findings showed that having contact with people with flu quadrupled the likelihood of the flu vaccine uptake (p=0.029). Strangely, having had flu and experiencing flu complications in the past were associated with lower influenza vaccine uptake (p=0.006 and p=0.033, respectively). This may be attributed to the false beliefs and myths surrounding the vaccine's efficacy. A previous study evaluating myths about flu vaccines found that 43% of participants believed that the vaccine could cause or exacerbate flu infection [32]. According to WHO, the five most common myths about flu vaccines are as follows: influenza is not so serious that it needs vaccines; flu vaccines cause flu; flu vaccines cause severe adverse effects; flu vaccines do not work; and pregnant women should not take flu vaccines [33]. Since individuals' perception of vaccine effectiveness influences their decision to get vaccinated, addressing misconceptions and myths surrounding flu vaccines and providing clear evidence of the vaccine's effectiveness in reducing illness severity, hospitalizations, and complications can positively impact vaccine uptake.

Safety concerns have been reported by other previous studies to be associated with low vaccine acceptance [12,13,32], aligning with our study findings showing a significant association between perceiving potential risks of flu vaccine and a lower likelihood of taking it (aOR: 0.213, p=0.042). Transparency in communication, addressing safety concerns promptly, and adopting a patient-centered approach can help build trust. This can be done by involving community leaders, healthcare providers, and public health experts to facilitate collaboration and instill confidence in the flu vaccine. Campaigns and advertisements constitute another option that can be effective, as our findings showed that seeing an ad for the flu vaccine was associated with a five-fold higher likelihood of taking the vaccine (aOR: 5.488, p=0.042).

There are some limitations to consider when interpreting this study’s results. We used a cross-sectional design, which imposed limitations in determining a cause-and-effect link or examining behavior across time. This study was conducted online, which could have led to selection bias. The sample was not properly representative of the population in Saudi Arabia as most of the participants were from Riyadh. Hence, the results might not accurately reflect the situations in other provinces. The use of an Arabic questionnaire, the low proportion of non-Saudi participants, and the non-representation of illiterate people and those with no access to the internet limit the generalizability of the results to these populations. Additionally, the proportion of participants with high education levels (23.8%) is not representative of the education level of the general population, limiting the generalizability of results. Around a third of the participants worked in the healthcare sector, which might influence the results in a way it might have overestimated the overall acceptance of flu vaccines. Therefore, more extensive longitudinal or experimental studies involving all population groups are recommended to mitigate these limitations.

## Conclusions

This study showed a lower flu vaccine uptake among the participants, which is consistent with previous studies. The determinants identified included sociodemographic factors, such as gender and work experience. Influenza infection-related psychosocial factors identified were contact with people having flu, having flu in the past, and experiencing severe flu complications, while vaccine-related psychosocial factors included perceiving potential risks and seeing an ad for the flu vaccine. Addressing these determinants can lead to a higher flu vaccine uptake and improve public health outcomes. A comprehensive approach should focus on improving healthcare accessibility, implementing more health education and awareness campaigns, addressing cultural and religious concerns, building trust in vaccines, emphasizing the vaccine's effectiveness, and implementing supportive policies and workplace initiatives. Mandatory vaccination requirements for specific groups, such as healthcare workers, can improve overall vaccine coverage. Additionally, workplace vaccination programs and incentives can encourage employees to get vaccinated, thereby reducing the risk of flu transmission.
